# How woodcocks produce the most brilliant white plumage patches among the birds

**DOI:** 10.1098/rsif.2022.0920

**Published:** 2023-03-01

**Authors:** Jamie Dunning, Anvay Patil, Liliana D'Alba, Alexander L. Bond, Gerben Debruyn, Ali Dhinojwala, Matthew Shawkey, Lukas Jenni

**Affiliations:** ^1^ Department of Life Sciences, Imperial College London, London, UK; ^2^ School of Polymer Science and Polymer Engineering, The University of Akron, Akron, OH, USA; ^3^ Department of Biology, Evolution and Optics of Nanostructure Group, University of Ghent, Gent 9000, Belgium; ^4^ Bird Group, The Natural History Museum, Tring, UK; ^5^ Swiss Ornithological Institute, Sempach, Switzerland; ^6^ Naturalis Biodiversity Center, Leiden, The Netherlands; ^7^ CertainTeed LLC, Malvern, PA, USA

**Keywords:** electron microscopy, reflectance, *Scolopax*, spectrophotometry, visual communication, finite-difference time-domain modelling

## Abstract

Until recently, and when compared with diurnal birds that use contrasting plumage patches and complex feather structures to convey visual information, communication in nocturnal and crepuscular species was considered to follow acoustic and chemical channels. However, many birds that are active in low-light environments have evolved intensely white plumage patches within otherwise inconspicuous plumages. We used spectrophotometry, electron microscopy, and optical modelling to explain the mechanisms producing bright white tail feather tips of the Eurasian woodcock *Scolopax rusticola*. Their diffuse reflectance was approximately 30% higher than any previously measured feather. This intense reflectance is the result of incoherent light scattering from a disordered nanostructure composed of keratin and air within the barb rami. In addition, the flattening, thickening and arrangement of those barbs create a Venetian-blind-like macrostructure that enhances the surface area for light reflection. We suggest that the woodcocks have evolved these bright white feather patches for long-range visual communication in dimly lit environments.

## 1. Introduction

The use of contrasting plumage patches or complex feather structures to convey information is widespread in birds (reviewed in [1,2]). Unlike in diurnal birds, visual signals in nocturnal and crepuscular species are understudied, and communication was, until recently, considered to follow chemical and acoustic channels [3–5]. However, in dim light environments, plumage characteristics have emerged that maximize reflectance of available light [6,7]. While most nocturnal and crepuscular birds have inconspicuous or cryptic plumages, visual signals are typically intensely white; for example, the white patches in the plumage of some nightjars Caprimulgidae [8], true owls Strigidae [9–11], stone-curlews Burhinidae [12] and snipes Scolopacidae [13].

The function and the mechanism by which these white patches optimize light reflectance is not well understood (but see [14,15]), but they probably communicate intention, for example, mating or territorial behaviours or signal quality ([13]; but also see [16]). However nocturnal and crepuscular birds typically also require crypsis while roosting during daylight [17,18] and therefore conceal their visual signals. White wing patches of some nightjars are, for example, only exposed in flight [8]; Or, in the woodcocks *Scolopax* spp, white undertail feather patches are only exposed when the tail is raised, or when engaging in roding display flights ([19]; figure 2*a*).

Borodulina & Formosow [19] first described modifications to the rami (radiating from the central rachis of the feather) that comprise the white tips on the underside of the tail feathers (hereafter rectrices) of the Eurasian woodcock *Scolopax rusticola* (hereafter woodcock; figure 2*a*) but did not measure reflectance and characterize its mechanism. Previous studies have demonstrated how microstructures correlate with white plumage intensity, for example in the winter body plumage of the rock ptarmigan *Lagopus muta* [20], the opal-like colours on some manakin birds Pipridae [14] and between many white-plumaged birds from different families [15]. Likewise, ‘super-white’, derived of microstructures on the carapace of a beetle [21,22] were well reported. The white patches in nocturnal and crepuscular birds, which are potentially optimized for signalling in low-light conditions, have seldom been addressed and require more detailed analysis.

Here we describe the mechanisms by which the white rectrix tips of the woodcock produce an intense white signal in low light conditions, using angle-resolved and diffuse spectrophotometry, electron microscopy and optical modelling via finite-difference time-domain (FDTD) approaches.

## 2. Material and methods

### 2.1. Microscopy

To characterize the microstructure and nanostructure responsible for producing the bright white signal, we used scanning and transmission electron microscopy (SEM and TEM, respectively). For SEM, we mounted individual white and brown rami (obtained from the same feather) separately, on stubs with carbon tape. We also oriented small fragments of rami in a way that allowed their observation in cross section. We sputter-coated the samples with gold/palladium for 2 min and imaged them on a SEM (FlexSEM 1000; Hitachi) at an accelerating voltage of 10 kV and 6 mm working distance.

For TEM we first embedded individual rami following a standard protocol [23]. Briefly, we rinsed and dehydrated the rami using ethanol three times, and then infiltrated them with increasing concentrations (15%, 50%, 70% and 100%) of epoxy resin (EMbed-812; Electron Microscopy Sciences, PA, USA) followed by 16 h polymerization in epoxy resin at 60°C in a laboratory oven.

We trimmed the blocks containing the rami and cut 100 nm thick cross sections using a Leica UC-6 ultramicrotome (Leica Microsystems, Germany). We collected the sections using oval-slit carbon and formvar-coated copper grids in duplicate and stained with Uranyless/lead citrate. We observed the sections on a JEOL JEM 1010 (Jeol Ltd, Tokyo, Japan) transmission electron microscope operating at 120 kV.

### 2.2. Spectrophotometry

We used micro- and (macro)spectrophotometry to measure reflectance spectra from three separate rectrices. We used the same feathers, but from different individual birds for all analyses. We measured reflectance from the reverse (under side) surface of a white ramus using a micro-spectrophotometer (CRAIC AX10: sensitivity 320–800 nm), and a spectrophotometer that measured a region across several rami (approx. 2 mm spot size). We measured diffuse (all reflected light) and specular reflectance (light reflected at a specific angle) between 300 and 700 nm in increments of 1 nm using an AvaSpec-2048 spectrometer and dual light source set-up (AvaLight-DH-S deuterium-halogen light source and AvaLight-HAL-S-MINI light source). We measured diffuse reflectance (which assumes that light reflectance is influenced by internal structures as well as those on an object's surface) using a bifurcated probe and an integrating sphere with a black gloss trap to exclude specular (light reflected from an objects surface) reflectance (AvaSphere-50-REFL). Then, we measured specular reflectance at three different angles (75°, 60°, 45°) using a bifurcated probe and a block holder (AFH-15, Avantes). We placed each feather on black paper minimizing background reflectance. All measurements are expressed relative to a 99% white reflectance standard (WS-2, Avantes) and 2% Avantes black standard (BS-2, Avantes). We processed data in the R package pavo in R 4.1.2 [24,25] and plotted them with previously published measurements from 61 other birds using identical spectrophotometric methods [15].

### 2.3. Finite-difference time-domain simulations

To explore the directionality of reflectance as a function of varying rami angle, we modelled how photons interact with structures within an individual barb. We ran a series of finite-difference time-domain (FDTD) simulations using a commercial-grade Ansys Lumerical 2021 R1 solver (Ansys, Inc.). The FDTD method provides a general solution to any light-scattering problem on complex arbitrary geometries (in this case, a computer aided design (CAD)-rendered ‘unit cell’ structure of an individual ramus) by numerically solving Maxwell's curl equations on a discrete spatio-temporal grid [26]. The simulation estimates all scattered light at a specific incident angle of incidence (hereafter, AOI). These simulated results can be compared with the diffuse spectrophotometry data.

**Figure 1.**
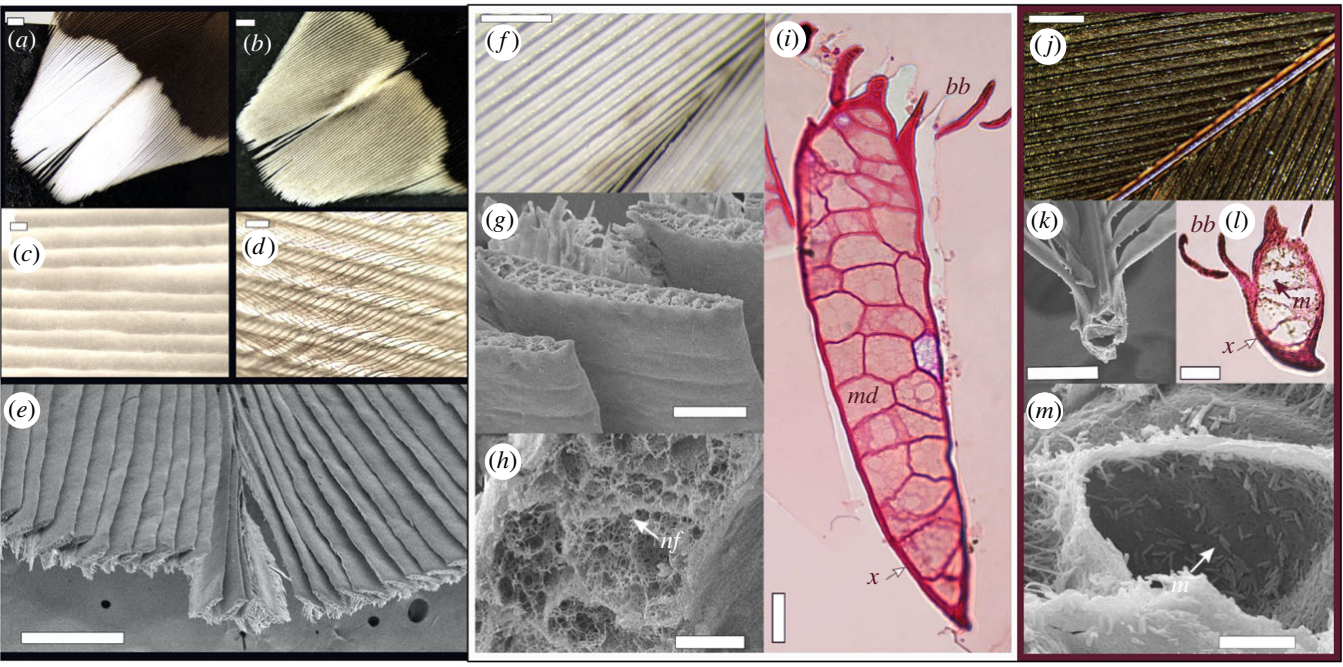
(*a*)–(*e*): Morphology of the white tips of woodcock *Scolopax rusticola* rectrices. (*a*) White reverse surface. (*b*) Brown obverse surface. (*c*) White rami in a Venetian-blind alignment; individual cells are apparent. (*d*) Obverse view showing the interlocked dark barbules covering the white rami. (*e*) SEM micrograph of the white rectrix tip transversally cut, showing shallow V-shaped surface of rami; (*f*)–(*m*): Comparison of the microstructure of the white and brown parts of rectrices. (*f*) Optical image of white rami at 30x magnification. (*g*) Thickened and flattened rami viewed from the reverse surface. (*h*) Interior of a white ramus shows cells with networks of keratin fibres (*nf*) and air pockets. (*i*) a white ramus showing hollow medullary cells (*md*) and a thin cortex (*x*); the barbules (*bb*) are present on the obverse side. (*j*) Optical image of contiguous brown region at 30x magnification. (*k*) Brown rami in cross-section. (*l*) Melanosomes (*m*) present throughout the rami and barbules. (*m*) Medullary cell of brown ramus showing melanosomes (*m*) and the absence of keratin matrices. Scale bars: (*a*) and (*b*) 1 mm; (c), (*d*), (*g*) and (*k*) 50 μm; (*e*) 500 μm; (*h*) 10 μm; (*i*) and (*l*) 100 μm; (*m*) 5 μm; (*f*) and (*j*) 1 mm.

Our simulated three-dimensional CAD models were based on empirical microscopic observations of the woodcock barbs (see electronic supplementary material, S1:A–D). First, we rendered a three-dimensional CAD geometry for a control hollow unit cell, without internal photonic nanostructures (in this case, the geometry without the structures observed in figure 1*h*) and a solid control unit cell.

We used SEM microscopy to define CAD dimensions and defined each unit cell by a keratin cortex thickness of 7 µm with a hollow interior, 20 µm high (Z direction) and 8 µm wide (X direction). We then used SEM microscopy to render an analogous simple unit cell (hereafter unit cell) with an internal nanostructure equivalent to the woodcock's rami, i.e. of air pockets and a supporting matrix of nano-fibres (figure 1). We did this using a uniform random distribution of non-overlapping spherical particles within the keratin matrix, which randomly varied in diameter between 0.45 and 3.45 µm. The optical constants (complex refractive indices) for keratin were adapted from previous literature ([27]; electronic supplementary material, table S1).

We performed simulations using a broadband plane wave source (400–700 nm), propagated along the −Z direction. First, at a normal (0°) AOI and then at 70° from surface normal, for our control, hollow and solid, CAD-rendered unit cells. Then, we ran simulations using our simulated woodcock unit cell at 0° and at 20°, 50°, 70° and 80° AOI from surface normal. Boundary conditions in the lateral direction (X and Y) were set to periodic. We monitored reflectance data using a discrete fourier transform (DFT) power monitor placed behind the source injection plane. The simulation time (in fs) and boundary condition along the light propagation direction (Z; perfectly matching layer (PML) boundaries) were chosen such that the electric field decayed before the end of the simulation (auto-shut-off criteria). All the incident light was either reflected, transmitted or absorbed.

### 2.4. Prevalence in related taxa

**Figure 2.**
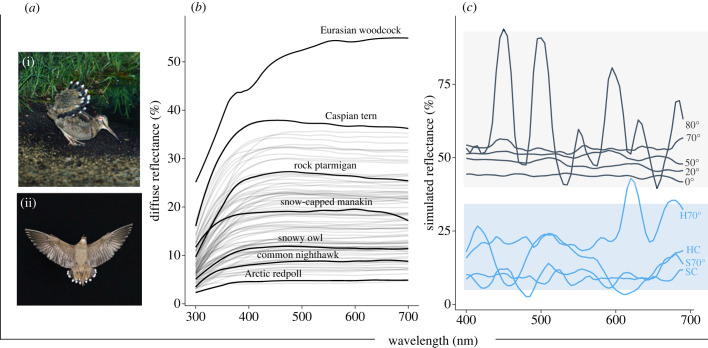
(*a*) Eurasian woodcock *Scolopax rusticola* showing ecological context when white tips are exposed, either from the ground (probably a female attracting an overflying male) (*a*(i)) or in flight (male in display flight) (*a*(ii)); photos by Serge Santiago and Jean-Lou Zimmermann. (*b*) Diffuse reflectance spectra measured from the reverse surface of the white Eurasian woodcock rectrix tips, peaking at approximately 55%, 31% brighter than the next brightest feather, Caspian tern *Hydroprogne caspia*, and compared against 61 white plumages from Igic *et al.* [15], species mentioned in text are highlighted; (*c*) finite-difference time-domain (FDTD) simulations showing simulated reflectance at five angles of incidence from a CAD rendered unit cell representing the tip of a woodcock tail feather (AOI; highlighted in grey, 0° (surface normal), 20°, 50°, 70° and 80°) and two control unit cells, measured at AOI (highlighted in blue; a hollow unit cell at 70° (H70°), a hollow control at surface normal (HC), solid control at 70° (S70°) and solid control at surface normal (SC)). These data suggest that air pockets present in the keratin matrix are essential for increasing the reflectance across visible wavelengths in the woodcock's tail feathers.

Finally, we examined specimens of eight species of Woodcock (including *S. rusticola*) and twenty-three species of closely related non-*Scolopax* Scolopacidae in museum collections for white tail feather tips, matching those described here (as figure 2*a*; see Acknowledgements for a list of institutions).

## 3. Results

### 3.1. Structure of the white rectrix tips

The tips of the rectrices are white on the reverse (figures 1*a* and 2*a*), but greyish brown on the obverse (upper side) surface (figure 1*b*). The rami are thickened and flattened in the white patch and overlap each other, superficially like Venetian blinds (figure 1*c,e*). The angle of these rami relative to the feather surface vary (as suggested in [19]), we estimated from approximately 70° for proximal rami to approximately 76° for distal rami (figure 1*e*). The proximal and distal brown barbules originate from the upper surface of the rami, hence are only visible on the obverse surface and cover the thickened white rami from above, providing the greyish brown colour of the obverse surface (figure 1*b*,*d*). They interlock to form a coherent vane. The two sides of a white tip, separated by the rachis, are concave and the barbs arranged in opposite angles (figure 1*j*), reflecting light in different directions and apparent when turning a feather in low light. By contrast, the brown parts of the rectrices are structurally typical of vaned feathers with thin barbs that are spaced by the brown barbules (figure 1*j*,*k*). The thickened white rami in the feather tips were approximately 2.5 times thicker and appeared internally more complex than brown rami (figure 1*f–h* and *j–m*, respectively). The medulla of white rami contained numerous and complex photonic cells with fine networks of nanofibres and scattered air pockets (figure 1*g–i*), lacking melanosomes entirely. These matrices of air and keratin appeared disorganized. By contrast, rami from brown feather regions were less thick, rounder, had fewer medullary cells and did not contain a matrix of air and keratin, but were abundant in melanosomes both inside the barb medulla and the cortex (figure 1*k–m*).

### 3.2. Reflectance

Spectrophotometry revealed intense diffuse reflectance across rami on the white underside of the rectrices, peaking at 55% (628 nm) (figures 1*f* and 2*a*). Likewise, individual rami had even greater specular reflectance, peaking greater than 100% against a diffuse standard (electronic supplementary material, figure S2). The white patches on woodcock rectrices are therefore exceptionally bright, and, to the best of our knowledge, represent the brightest white measured from the plumage of a bird, 31% brighter than the next most reflective, Caspian tern *Hydroprogne caspia*, that peaks at 38% (459 nm), and 91% brighter than the least-reflective white feather measured, Arctic redpoll *Acanthis hornemanni*, that peaks at 4.9% (638 nm) ([15]; figure 2*b*). Specular reflection was highest when measured at 75° relative to surface normal, decreasing at more acute angles (electronic supplementary material, figure S3).

### 3.3. Finite-difference time-domain simulations of reflectance

We found the disordered nanostructure formed by keratin and air phases in the woodcock rami were essential for generating intense white reflectance. For normal incidence, the overall reflectance (integrated across all angles) for the woodcock-mimicked rami unit cell nanostructure increased by approximately 65% with respect to the control hollow unit cell nanostructure. Additionally, the simulations also highlight some directionality to patch intensity. Modelled reflectance at 80° resulted in increased oscillation in spectra, derived of constructive and destructive interference. Otherwise, the reflectance increased from a peak of approximately 45% at normal incidence, to a peak of approximately 57% at 70°, which represents the actual angle of the rami within the white patch (figure 2*c*). Modelled reflectance at 75° (not shown in figure 2*c* but see [28]; electronic supplementary material, figure S4) and 80° AOI showed increasing noise, which we suggest is due to interference effects (highlighted by increasing standard deviations around the mean reflectance as AOI increased; electronic supplementary material, figure S4). Reflectance at 70° is broadly the same as the actual diffuse reflectance (figure 2*c*), although FDTD simulates diffuse plus specular reflectance. We, therefore, suggest that the rami are arranged to lie at the angle which best optimizes reflectance. Further, our simulated control unit cells demonstrate that air pockets in the keratin matrix are essential for increasing the overall reflectance across visible wavelengths.

### 3.4. Prevalence in related taxa

We recorded equivalent white patches, defined by the presence of thick and flattened white rami on the tail feather tips in all eight species of woodcock, but not in their closest relatives (23 species of non-*Scolopax* Scolopacidae, see electronic supplementary material, table S1).

## 4. Discussion

Our results suggest that the white tips on the woodcock's rectrices represent the brightest reflectance yet measured and, by virtue, the whitest white plumage patch currently known among the birds. Although intense reflectance in white plumages have been reported previously [20,29,30], these were without standardized comparison to other white-plumaged species. Thus, we present our results alongside those previously described plumages (see [15] for a full list), using standardized spectrophotometry methods (figure 2*b*). This reflection is produced by the arrangement of thick and flattened rami with a broad distribution of air pockets, that together maximize light reflection intensity. We used FDTD simulations to demonstrate that (i) the internal structure of the rami on the white tips is integral for light scattering and subsequent reflection intensity, but also (ii) that the angle of the broadened barbs in relation to each other optimize reflectance at the macro-scale.

The structures we describe differ from those of less intensely reflective white plumages in two ways: first, the rami are thickened and flattened ([19]; this study), increasing surface area available for reflection and preventing light from passing between the rami and barbules. Second, the thickened rami allow for a complexity of photonic cells, with a network of keratin nanofibres and scattered air pockets, creating numerous interfaces to favour scattering events (like the ‘super-white’ reflectance described in a white beetle; [21,22]).

Igic *et al.* [15] suggested that more intense reflectance of white plumage was associated with densely packed, rounder and less hollow rami, but also thicker and longer barbules. Consequently, larger species were brighter by virtue of rami thickness and complexity. However, the woodcock rami are thickened and flattened, superficially like the rami in the white crown of blue-rumped manakin *Lepidothrix isidorei* [14]; in this case, the internal nanostructure is without the thickened rami that increases the surface area of reflection. Despite some similarities, the diffuse reflectance of the manakin's crown peaks at approximately 17% [14], approximately 105% less bright than the woodcock. However, specular reflectance of the manakin crown is higher than the woodcock, due to a nanostructure that enhances specular reflectance (also see [31,32]). The Venetian-blind arrangement of the thickened rami, and subsequent directional reflection, is superficially like the arrangement of barbules of some hummingbirds Trochilidae. In this case, the angle of the barbules relative to the axis of the ramus, and the angle between the proximal and distal barbules of the rami determine directionality of reflectance, associated with irradiance [33], similar to the mechanism we have described in the woodcock tail feather tip.

White patches are present in all eight species of woodcock, but not in their closest relatives (23 species of non-*Scolopax* Scolopacidae, see electronic supplementary material, table S1). We did not undertake spectrophotometry measurements nor microscopy work on any woodcock taxa, beyond *S. rusticola*, and so could not compare their respective reflectance spectra.

We suggest that white patches present in the woodcocks tail feathers are linked to signalling some behaviour in dimly lit environments [12,34]. Because these patches are only visible from below, any functional significance is conditional on raising and fanning the tail, for example during courtship displays [35–38], predator distraction or non-reproductive communication [39,40]. The link between patch intensity, behaviour and relative light environment is understudied and would benefit from further research.

We suggest that the woodcocks have evolved brilliant white feather patches, the brightest described within the birds, through elaborate structural modifications at the macro-, micro- and nano scales for communication in dimly lit environments.

## Data Availability

Original data collected as part of this study has been made available from the Dryad Digital Repository [28]. The data are provided in electronic supplementary material [41].

## References

[1] Jenni L, Winkler R. 2020 The biology of moult in birds. London, UK: Helm.

[2] Terril RS, Shultz AJ. 2022 Feather function and the evolution of birds. Biol. Rev. (10.1111/brv.12918) 36424880

[3] Healy S, Guilford T. 1990 Olfactory-bulb size and nocturnality in birds. Evolution **44**, 339-346. (10.2307/2409412) 28564375

[4] Bonadonna F, Bretagnolle V. 2002 Smelling home: a good solution for burrow-finding in nocturnal petrels? J. Exp. Biol. **205**, 2519-2523. (10.1242/jeb.205.16.2519) 12124375

[5] Grieves LA, Gilles M, Cuthill IC, Székely T, MacDougall-Shackleton EA, Caspers BA. 2022 Olfactory camouflage and communication in birds. Biol. Rev. **97**, 1193-1209. (10.1111/brv.12837) 35128775

[6] Endler JA. 1993 The color of light in forests and its implications. Ecol. Monogr. **63**, 1-27. (10.2307/2937121)

[7] Penteriani V, Del Mar Delgado M. 2017 Living in the dark does not mean a blind life: bird and mammal visual communication in dim light. Proc. R. Soc. B **372**, 20160064. (10.1098/rstb.2016.0064) PMC5312014 28193809

[8] Aragonés J, Arias De Reyna L, Recuerda P. 1999 Visual communication and sexual selection in a nocturnal bird species, *Caprimulgus ruficollis*, a balance between crypsis and conspicuousness. Wilson Bull. **111**, 340-345.

[9] Penteriani V, Del Mar Delgado M, Alonso-Alvarez C, Sergio F. 2007 The importance of visual cues for nocturnal species: eagle owls signal by badge brightness. Behav. Ecol. **18**, 143-147. (10.1093/beheco/arl060)

[10] Bortolotti GR, Stoffel MJ, Galván I. 2011 Wintering snowy owls *Bubo scandiacus* integrate plumage colour, behaviour and their environment to maximize efficacy of visual displays. Ibis **153**, 134-142. (10.1111/j.1474-919X.2010.01067.x)

[11] Bettega C, Campioni L, Del Mar Delgado M, Lourenço R, Penteriani V. 2013 Brightness features of visual signalling traits in young and adult Eurasian eagle-owls. J. Raptor Res. **47**, 197-207. (10.3356/JRR-12-00002.1)

[12] Cramp S, Simmons KEL. 1983 Handbook of the birds of Europe, the Middle East and North Africa. Vol 3: The birds of the Western Palearctic. Oxford, UK: Oxford University Press.

[13] Höglund J, Eriksson M, Lindell LE. 1990 Females of the lek-breeding great snipe, *Gallinago media*, prefer males with white tails. Anim. Behav. **40**, 23-32. (10.1016/S0003-3472(05)80662-1)

[14] Igic B, D'Alba L, Shawkey MD. 2016 Manakins can produce iridescent and bright feather colours without melanosomes. J. Exp. Biol. **219**, 1851-1859. (10.1242/jeb.137182) 27307543

[15] Igic B, D'Alba L, Shawkey MD. 2018 Fifty shades of white: how white feather brightness differs among species. Sci. Nat. **105**, 3-4. (10.1007/s00114-018-1543-3) 29445955

[16] Sæther SA, Fiske P, Kålås JA, Gjul JM. 2000 Females of the lekking great snipe do not prefer males with whiter tails. Anim. Behav. **59**, 273-280. (10.1006/anbe.1999.1301) 10675249

[17] Troscianko J, Wilson-Aggarwal JK, Stevens M, Spottiswoode CN. 2016 Camouflage predicts survival in ground-nesting birds. Sci. Rep. **6**, 19966. (10.1038/srep19966) 26822039 PMC4731810

[18] Stevens M, Troscianko J, Wilson-Aggarwal JK, Spottiswoode CN. 2017 Improvement of individual camouflage through background choice in ground-nesting birds. Nat. Ecol. Evol. **1**, 1325-1333. (10.1038/s41559-017-0256-x) 28890937 PMC5584661

[19] Borodulina TL, Formosow AN. 1967 About signal spots of feathering of birds and peculiarity of woodcock rectrices (in Russian). Bjull. Mosk. obstsch. ispyt. prirody, otd. biol. **72**, 27-31. Also published in *Russkii Ornitologicheskii Zhurnal* 24. 2015. Ekspress Vypusk 1230: 4622–4626.

[20] Dyck J. 1979 Winter plumage of the rock ptarmigan: structure of the air-filled barbules and function of the white colour. Dansk. Orn. Foren. Tidsskr. **73**, 41-58.

[21] Vukusic P, Hallam B, Noyes J. 2007 Brilliant whiteness in ultrathin beetle scales. Science **315**, 348. (10.1126/science.1134666) 17234940

[22] Burresi M, Cortese L, Pattelli L, Kolle M, Vukusic P, Wiersma DS, Steiner U, Vignolini S. 2014 Bright-white beetle scales optimise multiple scattering of light. Sci. Rep. **4**, 6075. (10.1038/srep06075) 25123449 PMC4133710

[23] D'Alba L, Meadows M, Maia R, Jong-Souk Y, Manceau M, Shawkey M. 2021 Morphogenesis of iridescent feathers in Anna's hummingbird *Calypte anna*. Integr. Comp. Biol. **61**, 1502-1510. (10.1093/icb/icab123) 34104966

[24] Maia R, Gruson H, Endler JA, White TE. 2019 pavo 2: new tools for the spectral and spatial analysis of colour in R. Methods Ecol. Evol. **10**, 1097–-1107. (10.1111/2041-210x.13174)

[25] R Core Team. 2022 R: A language and environment for statistical computing. Vienna, Austria: R Foundation for Statistical Computing. See https://www.R-project.org/.

[26] Taflove A, Hagness SC. 2005 Computational electrodynamics: the finite-difference time-domain method, 3rd edn. Norwood, MA: Artech House, Inc.

[27] Stavenga DG, Leertouwer HL, Osorio DC, Wilts BD. 2015 High refractive index of melanin in shiny occipital feathers of a bird of paradise. Light Sci. Appl. **4**, e243. (10.1038/lsa.2015.16)

[28] Dunning J, Patil A, D'Alba L, Bond AL, Debruyn G, Dhinojwala A, Shawkey M, Jenni L. 2023 Data from: How woodcocks produce the most brilliant white plumage patches among the birds. Dryad Digital Repository. (10.5061/dryad.31zcrjdqs) PMC9974297 36854381

[29] Tickell WLN. 2003 White plumage. Waterbirds: Int. J. Waterbird Biol. **26**, 1-12.

[30] Stoddard MC, Prum RO. 2011 How colorful are birds? Evolution of the avian plumage color gamut. Behav. Ecol. **22**, 1042-1052. (10.1093/beheco/arr088)

[31] Shawkey MD, Maia R, D'Alba L. 2011 Proximate bases of silver color in anhinga (*Anhinga anhinga*) feathers. J. Morphol. **272**, 1399-1407. (10.1002/jmor.10993) 21755527

[32] McCoy DE, Shultz AJ, Vidoudez C, van der Heide E, Dall JE, Trauger SA, Haig D. 2021 Microstructures amplify carotenoid plumage signals in tanagers. Sci. Rep. **11**, 1-20. (10.1038/s41598-020-79139-8) 33883641 PMC8060279

[33] Giraldo M, Sosa J, Stavenga D. 2021 Feather iridescence of *Coeligena* hummingbird species varies due to differently organized rami and barbules. Biol. Lett. **17**, 20210190. (10.1098/rsbl.2021.0190) 34428957 PMC8385349

[34] Glutz Von Blotzheim UN, Bauer KM, Bezzel E. 1977 Handbuch der vögel mitteleuropas, vol 7. Leipzig, Germany: Akademische Verlagsgesellschaft.

[35] Hagen Y. 1950 How a woodcock (*Scolopax rusticola*) sitting on the ground attracts the attention of partners in mating flight. Vår Fågelvärld **9**, 195-199.

[36] Hirons G. 1980 The significance of roding by woodcock *Scolopax rusticola*: an alternative explanation based on observations of marked birds. Ibis **122**, 350-354. (10.1111/j.1474-919X.1980.tb00888.x)

[37] Ferrand Y, Gossmann F. 2009 La bécasse des bois. Saint-Lucien, France: Effet de lisière éditeur.

[38] Lastukhin AA, Isakov AM. 2016 Reflection of light from white spots on the tail of a woodcock *Scolopax rusticola* in flight (in Russian). Russkii Ornitologicheskii Zhurnal 25, Ekspress Vypusk. **1312**, 2642-2643.

[39] Ingram C. 1974 Possible functions of the tail spots in the woodcock. Brit. Birds **67**, 475-476.

[40] Fetisov CA. 2017 On the functional significance of bright white spots on the tail of the woodcock *Scolopax rusticola* (in Russian). Russkii Ornitologicheskii Zhurnal 26, Ekspress Vypusk. **1466**, 2727-2733.

[41] Dunning J, Patil A, D'Alba L, Bond AL, Debruyn G, Dhinojwala A, Shawkey M, Jenni L. 2023 How woodcocks produce the most brilliant white plumage patches among the birds. Figshare. (10.6084/m9.figshare.c.6430381) PMC9974297 36854381

